# Challenges to Sustainable Safe Drinking Water: A Case Study of Water Quality and Use across Seasons in Rural Communities in Limpopo Province, South Africa

**DOI:** 10.3390/w10020159

**Published:** 2018-02-07

**Authors:** Joshua N. Edokpayi, Elizabeth T. Rogawski, David M. Kahler, Courtney L. Hill, Catherine Reynolds, Emanuel Nyathi, James A. Smith, John O. Odiyo, Amidou Samie, Pascal Bessong, Rebecca Dillingham

**Affiliations:** 1 Department of Hydrology and Water Resources, University of Venda, Thohoyandou 0950, South Africa; John.Odiyo@univen.ac.za; 2 Department of Civil and Environmental Engineering, University of Virginia, Charlottesville, VA 22904, USA; kahlerd@duq.edu (D.M.K.); clh023@gmail.com (C.L.H.); cfr4ms@virginia.edu (C.R.); jas9e@virginia.edu (J.A.S.); 3 Department of Public Health Sciences, University of Virginia, Charlottesville, VA 22908, USA; etr5m@virginia.edu; 4 Division of Infectious Diseases & International Health, University of Virginia, Charlottesville, VA 22908, USA; rd8v@hscmail.mcc.virginia.edu; 5 Center for Environmental Research and Education, Duquesne University, Pittsburgh, PA 15282, USA; 6 School of Civil and Environmental Engineering, Georgia Institute of Technology, Atlanta, GA 30332, USA; 7 Department of Animal Science, University of Venda, Thohoyandou 0950, South Africa; emanuel.nyathi@univen.ac.za; 8 Department of Microbiology, University of Venda, Thohoyandou 0950, South Africa; samie.amidou@univen.ac.za (A.S.); pascal.bessong@univen.ac.za (P.B.)

**Keywords:** water quality, water resources, water-resources management, public health, drinking water treatment

## Abstract

Consumption of microbial-contaminated water can result in diarrheal illnesses and enteropathy with the heaviest impact upon children below the age of five. We aimed to provide a comprehensive analysis of water quality in a low-resource setting in Limpopo province, South Africa. Surveys were conducted in 405 households in rural communities of Limpopo province to determine their water-use practices, perceptions of water quality, and household water-treatment methods. Drinking water samples were tested from households for microbiological contamination. Water from potential natural sources were tested for physicochemical and microbiological quality in the dry and wet seasons. Most households had their primary water source piped into their yard or used an intermittent public tap. Approximately one third of caregivers perceived that they could get sick from drinking water. All natural water sources tested positive for fecal contamination at some point during each season. The treated municipal supply never tested positive for fecal contamination; however, the treated system does not reach all residents in the valley; furthermore, frequent shutdowns of the treatment systems and intermittent distribution make the treated water unreliable. The increased water quantity in the wet season correlates with increased treated water from municipal taps and a decrease in the average contaminant levels in household water. This research suggests that wet season increases in water quantity result in more treated water in the region and that is reflected in residents’ water-use practices.

## 1. Introduction

Clean and safe drinking water is vital for human health and can reduce the burden of common illnesses, such as diarrheal disease, especially in young children. Unfortunately, in 2010, it was estimated that 1.8 billion people globally drank water that was not safe [1]. This scenario is most common in developing countries, and the problem is exacerbated in rural areas [1]. Significant amounts of time are spent by adults and school children upon water abstraction from various sources [2,3]. It is estimated that, in developing countries, women (64%) and girls (8%) spend billions of hours a year collecting water [1]. The erratic supply of safe drinking and domestic water often affects good hygiene practices. In most developing countries of the world, inadequate supplies of drinking water can contribute to the underage death of children in the region [4–10].

Storage of collected water from rivers, springs, community stand-pipes, and boreholes is a common practice in communities that lack potable water supplies piped into their homes. Even when water is piped into the home, it is often not available on a continuous basis, and water storage is still necessary. Water is stored in various containers which include jerry cans, buckets, drums, basins and local pots [11–13]. It has been reported that when collection of water from sources of high quality is possible, contamination during transport, handling and storage and poor hygienic practices often results and can cause poor health outcomes [11,13–15].

South Africa is a semi-arid country that has limited water resources, and the provision of adequate water-supply systems remains a great challenge. In some of the major cities, access to clean and safe drinking water is comparable to what is found in other developed cities, but this is not the case in some cities, towns and most villages where there is constant erratic supply of potable water, and in some cases, there is no water supply system [16]. Although access to clean and safe drinking water is stipulated as a constitutional right for all South Africans in the country’s constitution [17,18], sustainable access to a potable water supply by millions of South Africans is lacking.

Residents of communities with inadequate water supply are left with no alternative other than to find local sources of drinking water for themselves. Rural areas are the most affected, and residents resort to the collection of water from wells, ponds, springs, lakes, rivers and rainwater harvesting to meet their domestic water needs [19–24]. Water from such sources is often consumed without any form of treatment [12,19,21]. However, these alternative sources of drinking water are often vulnerable to point and non-point sources of pollution and are contaminated frequently by fecal matter [5,19,25]. A report by the South African Council for Scientific and Industrial Research clearly showed that almost 2.11 million people in South Africa lack access to any safe water infrastructure. The consumption of water from such unimproved sources without treatment constitutes a major public health risk [26].

Consumption of contaminated drinking water is a cause of diarrheal disease, a leading cause of child mortality in developing countries with about 700,000 deaths of children under the age of 5 reported in 2011 [10,27]. In South Africa, diarrhea is one of the leading causes of death among young children, and this problem is worst in children infected with HIV (Human Immunodeficiency Virus).

The health risks associated with the consumption of unsafe drinking water are not only related to infectious diseases but also to other environmental components such as fluoride, arsenic, lead, cadmium, nitrates and mercury. Excessive consumption of these substances from contaminated drinking water can lead to cancer, dental and skeletal fluorosis, acute nausea, memory lapses, renal failure, anemia, stunted growth, fetal abnormalities and skin rashes [16,28]. Groundwater contamination with high arsenic concentrations have been reported in Bangladesh, and high fluoride concentrations have been reported in the drinking water from various provinces in South Africa [28–34].

Temporary seasonal variations have been reported to influence the levels of contaminants in various water sources differently. The key environmental drivers across the wet and dry seasons include: volume of water, flow, frequency of rainfall events, storm run-off, evaporation and point sources of pollution [35,36]. An increase in storm-water run-off within a river catchment may increase the level of contaminants due to land-use activities. Increased water volume could lead to a decrease in the concentration of contaminants due to the dilution effect. A low incidence of rainfall and high evaporation can cause a contaminant to concentrate in water. Very few water-quality parameters such as turbidity are expected to be higher in the wet season. Other parameters can vary depending on the key environmental drivers. There is paucity of data on the effect of change across seasons on water-use practices among household in rural areas of developing countries.

The geographic area for this study is located 35 km north of Thohoyandou, in Limpopo Province, South Africa. The area is primarily agricultural, such that water contamination by nitrates is a potential concern. In addition, mining operations in the area may contaminate water sources with heavy metals.

The significance of this study lies in the broad characterization of water-quality parameters that could affect human health, which is not restricted to microbiological analysis. In a rural community, the primary concern of drinking water is the microbiological quality of the water and chemical constituents are often considered not as problematic. This study was designed to evaluate a broad spectrum of water-quality constituents of natural water sources and household drinking water used by residents of rural communities in Limpopo Province. We also aimed to determine how water sources and collection practices change between dry and wet seasons within a one-year sampling period.

## 2. Materials and Methods

### 2.1. Study Design

A baseline census of 10 villages in the Thulamela Municipality of Limpopo Province was completed to identify all households in which there was at least one healthy child under 3 years of age in the household, the child’s caregiver was at least 16 years of age, and the household did not have a permanent, engineered water-treatment system. 415 households that met these eligibility criteria were enrolled for the purposes of a water-treatment intervention trial. The baseline assessment of water-quality and use practices is reported here. Caregivers of the child under 3 years of age were given a questionnaire concerning demographics, socioeconomic status, water-use practices, sanitation and hygiene practices, and perceptions of water quality and health. In addition, a sample of drinking water was taken from a random selection of 25% of the total enrolled households in the dry (June–August 2016) and wet seasons (January–February 2017). The participant population was sorted by community, as a surrogate for water supply, and one-third from each community was randomly selected by a random number generated within Microsoft Excel (Seattle, WA, USA), which was sampled. The protocol used was approved by the Research Ethics Committee at the University of Venda (SMNS/15/MBY/27/0502) and the Institutional Review Board for Health Sciences Research at the University of Virginia (IRB-HSR #18662). Written informed consent was obtained from all participants and consent documentation was made available in English and Tshivenda. The majority of the baseline surveys were conducted in the dry season (approximately April to October). Six-months later, follow-on surveys were conducted at the height of the wet season (approximately November to March; however, the height of the season in 2016–17 was January to March).

### 2.2. Regional Description of the Study Area

**Figure 1.**
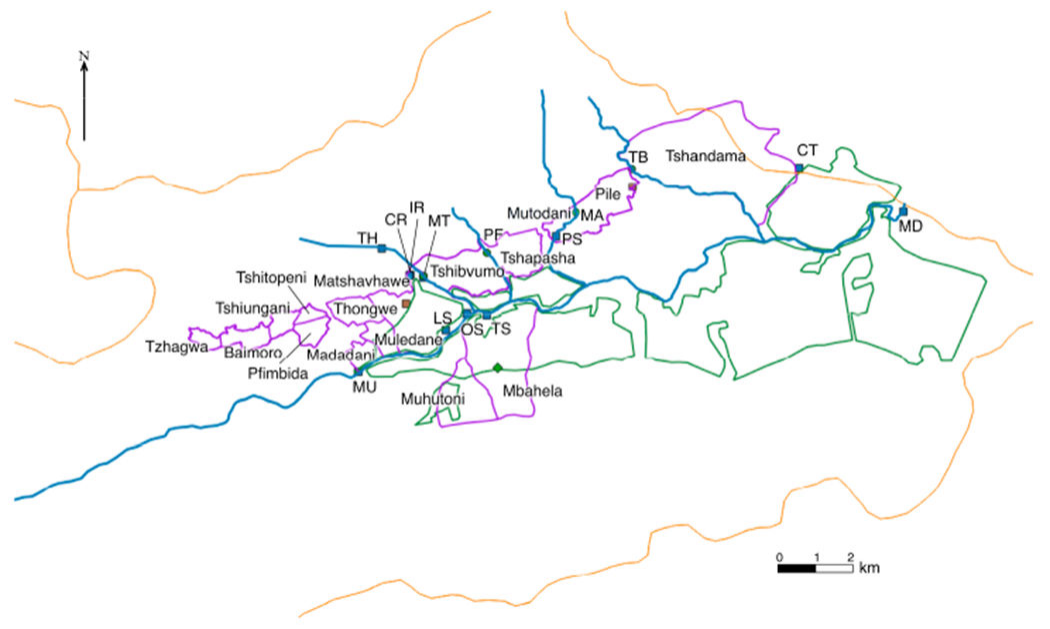
Map of the study area. The communities are all located within the Mutale River watershed. The rivers are indicated in blue, villages outlined in purple, environmental samples in blue squares, tributaries in green circles (which have intermittent flow), watershed boundary in orange. This heavily agricultural area has cultivated areas along both sides of thee Mutale River for the vast majority of the region; the area is shown with green outlines. There are two identified brick-processing areas shown in brown rectangles. Unfortunately, some sites are so close that the markers overlap (as with CR and IR). The location of the community supplies (CA, CB, and CC) are not shown to protect the privacy of those villages. See supplemental information for Google Earth files.

**Figure 2.**
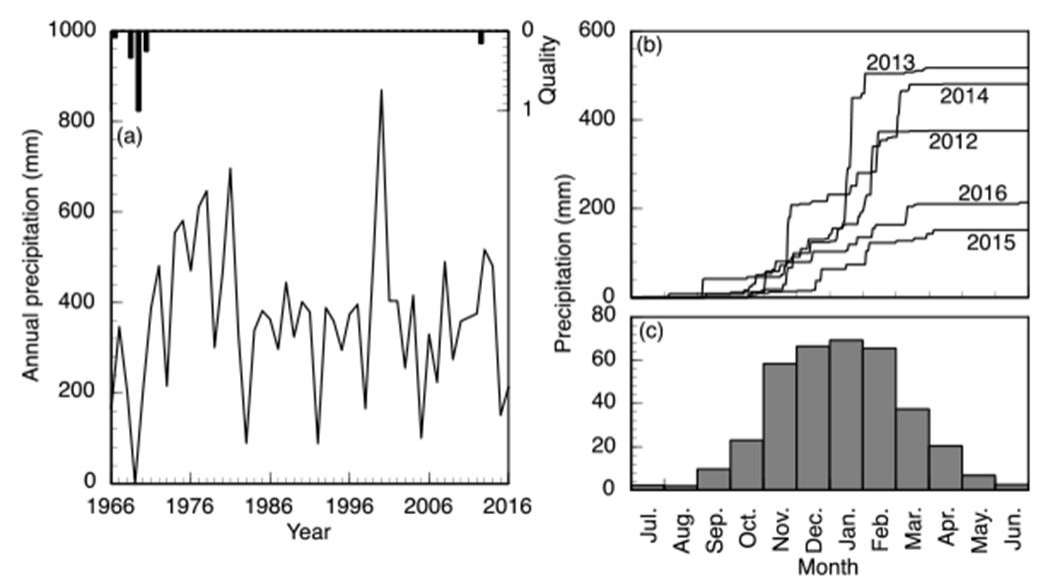
Precipitation trends in the study area. (**a**) Annual precipitation by hydrologic year. Data quality are presented on a scale of zero to unity where the quantity shown represents the proportion of missing or unreliable data in a year; (**b**) Cumulative precipitation for the last five complete years; (**c**) Average monthly precipitation calculated for years with greater than 90% reliable data (bottom right). All data are presented by the standard Southern hemisphere hydrologic year from July to June numbered with the ending year. Data are from the Nwanedzi Natural Reserve at the Luphephe Dam (17 km from the study area) and fire available through the Republic of South Africa, Department of Water and Sanitation, Hydrologic Services (http://www.dwa.gov.za/Hydrology/).

**Figure 3.**
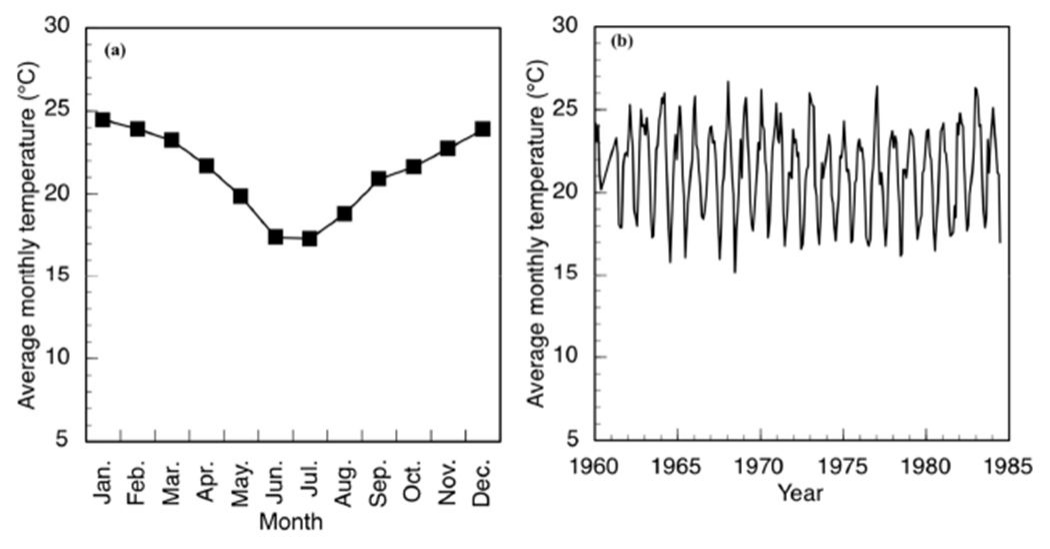
The mean monthly temperature in the region recorded at Punda Milia. (**a**) Mean monthly temperature based on the means from 1962–1984; (**b**) Mean monthly temperature record. Data are available from the National Oceanic and Aviation Administration (U.S.), National Climatic Data Center, Climate Data Online service (https://www.ncdc.noaa.gov/cdo-web/).

The communities are located in a valley in the Vhembe District of Limpopo Province, South Africa (Figure 1). The valley surrounds the Mutale River in the Soutpansberg Mountains and is located around 22°47′34′′ S and 30°27′01′′ E, in a tropical environment that exhibits a unimodal dry/wet seasonality (Figure 2). In recent years, the area has received annual precipitation between 400 mm and 1100 mm; more importantly, the timing of the precipitation is highly variable (Figure 2). Specifically, in 2010, the annual precipitation was about 750 mm; however, the majority of the precipitation came in March while, traditionally, the wet season begins earlier, in September or October. The year 2011 had the highest precipitation in the six-year period and had the majority of the rainfall in November. The years 2012 and 2015 began with a typical precipitation pattern; however, the rainfall did not continue as it did in 2013 and 2014. Annual temperature of the area also varies, with the highest temperature always recorded in the wet season (Figure 3). There has been much variability of temperature in past years; however, this is beyond the scope of this study. The abbreviations used in Figure 1 and other figures, including the supplementary data and the type of the various water sources used in this study, are shown in Table 1.

Agriculture occupies tine greatest land cover in the valley. Mogt households are engaged in some level of farming. Crops cultivated include maize and vegetables, and tree fruits include mangos and citrus fruits. Livestock is prevalent in the area with chickens, goats, and cattle. Smaller animals typically remain closer to households and larger animals graze throughout the region without boundaries. There are several brick-making facilities in the valley that include excavation, brick-forming and drying.

### 2.3. Water Sources

Drinking water in the study communities is available from a number of municipal and natural sources. The primary source of drinking water for seven of the villages is treated, municipal water. Two of the villages have community-level boreholes, storage tanks, and distribution tanks. An additional village has a borehole as well; however, residents report that, since its installation, the system has never supplied water.

The water for the treatment facility is drawn from behind a weir in the Mutale River and pumped to a retention basin. The water then undergoes standard treatment that includes pH adjustment, flocculation, settling, filtration, and chlorine disinfection. Water is then pumped to two elevated tanks that supply several adjacent regions, including the study area. Specifically, Branch 1 supplies Tshandama, Pile, Mutodani, Tshapasha and Tshibvumo; Branch 2 supplies an intermediary tank that in turn serves Matshavhawe, Muledane and Thongwe. Households can pay for a metered yard connection for the water used; these yard connections can be connected to household plumbing at the household’s discretion. The treated municipal water service is intermittent. Service in Tshandama and Pile was observed to be constant during the wet season and for only about two to three days per week during the dry season. Service in the remaining communities is two to four days per week during the wet season and about two days per week during the dry season. Furthermore, for the past two years, major repairs in the dry season caused the treated municipal water to cease completely. Households typically stored water for the periods when the treated municipal water was off; however, when the municipal water was unavailable for longer periods or not on the anticipated schedule, households obtained water from natural sources. The community-level boreholes provided water almost constantly but were subject to failure and delays in repairs.

Aside from the municipal sources, many residents of three villages have access to a community installed and operated distribution system that delivers water from the adjacent ephemeral rivers throughout the community (CA, CB, and CC). These systems are constructed with 50 mm to 70 mm (5 to 7 × 10^−2^ m) high-density polyethylene pipes. Even these community-level schemes provide water on a schedule and sometimes require repair. Another common source of water for the community is springs. These shallow groundwater sources are common in the valley; however, there are communities that do not have a nearby spring. Some springs have had a pipe placed at the outlet to keep the spring open and facilitate filling containers. Researchers did not observe any constructions around the springs to properly isolate them from further contamination, and they are, therefore, not improved water sources. Pit latrines are common in every household throughout the region. Source (TS) is located near these communities while other springs (OS, LS) are located in agricultural areas. Boreholes provide deep groundwater supplies but require a pump. Such systems provide water as long as there is power for the pump and the well is deep enough to withstand seasonal variations. The two clinics in the study area surveyed each relied on a borehole for their water supply. Some residents also collected water directly from the river. The Mutale River is a perennial river; however, the ephemeral rivers, the Tshiombedi, Madade, Pfaleni, and Tshala Rivers, do not flow in the dry season all the way to the floor of the valley. The Tshala River has a diversion to a lined irrigation canal that always carries water, but there is very little flow that remains in the natural channel.

### 2.4. Water Sampling

The team of community health workers (CHW) that had previously conducted the MAL-ED (Malnutrition and Enteric Diseases) study in the same region [37] were recruited to assist with the data collection for this study; specifically, the regional description and water sources. These CHWs have an intimate knowledge of the communities as they are residents and have conducted health research in the area. The CHWs provided information on the location and condition of the various water sources in the study communities.

Water sources were tested during two intensive study periods: one in the dry season (June–August, 2016) and the other in the wet season (January–February, 2017). Water sources for investigation were selected based on identification from resident community health workers. Single samples were taken from all 28 identified drinking water sources in the 10 villages and three days of repeated samples were taken from six sources, which represented a range of sources (e.g., surface, borehole, shallow ground, pond, and municipal treated) in the dry season. Single samples of 17 of the original sources and three days of repeated samples were taken from five sources in the wet season, six months later. Some sources were not resampled because the routes to the sources were flooded, and these sources were likely infrequently used during the wet season due to blocked pathways. The wet and dry season measurements gave two different scenarios for water-use behaviors and allowed the researchers to measure representative water-quality parameters.

### 2.5. Measurement of Physicochemical Parameters

Physicochemical parameters of source water samples were measured in the field by a YSI Professional Plus meter (YSI Inc., Yellow Springs, OH, USA) for pH, dissolved oxygen and conductivity. The probes and meter was calibrated according to the manufacturer’s instructions. Turbidity was measured in the field with an Orbeco-Hellige portable turbidimeter (Orbeco Hellige, Sarasota, FL, USA) (U.S. Environmental Protection Agency method 180.1) [38]. The turbidimeter was calibrated according to the manufacturer’s instructions. Measured levels were compared to the South African water-quality standards in the regulations [39], pursuant to the Water Services Act of 1997.

### 2.6. Microbiological Water-Quality Analysis

*Escherichia coli* (*E. coli*) and total coliform bacteria were measured in both source and household water samples by membrane filtration according to U.S. Environmental Protection Agency method 10,029 [40]. Sample cups of the manifold were immersed in a hot-water bath at 100 °C for 15 min. Reverse osmosis water was flushed through the apparatus to cool the sample cups. Paper filter disks of 47 mm (4.7 × 10^−2^ m) diameter and 0.45 μm (4.5 × 10^−7^ m) pore size (EMD Millipore, Billerica, MA, USA) were removed from their sterile, individual packages and transferred to the surface of the manifold with forceps with an aseptic technique. Blank tests were run with reverse osmosis dilution water. Two dilutions were tested: full-strength (100 mL sample) and 10^−2^ (1 mL sample with 99 mL of sterile dilution water) were passed through the filters; this provides a range of zero to 30,000 CFU/100 mL (colony forming units) for both *E. coli* and total coliforms. The filter paper was placed in a sterile petri dish with absorbent pad with 2 mL (2 × 10^−6^ m^3^) of selective growth media solution (m-ColiBlue24, EMD Millipore, Billerica, MA, USA). The samples were incubated at 35 °C (308.15 K) for 23–25 h. Colonies were counted on the full-strength sample. If colonies exceeded 300 (the maximum valid count), the dilution count was used. In all tests, the dilution value was expected to be within 10^−2^ of the full-strength value and the sample was discarded otherwise.

The distribution of the household bacteria levels was evaluated by the (chi square) χ^2^ goodness-of-fit test for various subsets of the data. Subsets of the data were then compared by an unpaired Student’s t-test for statistical significance; specifically, wet versus dry season levels as well as any other subsets that could demonstrate differences within the data.

### 2.7. Major Metals Analysis

A Thermo ICap 6200 Inductively Coupled Plasma Atomic Emission Spectrometer (ICP-AES, Chemetix Pty Ltd., Johannesburg, South Africa) was used to analyze the major metals in the various samples. The National Institute of Standards and Technology traceable standards (NIST, Gaithersburg, MD, USA) purchased from Inorganic Ventures (INORGANIC VENTURES 300 Technology Drive Christiansburg, Christiansburg, VA, USA) were used to calibrate the instrument for the quantification of selected metals. A NIST-traceable quality control standard from De Bruyn Spectroscopic Solutions, Bryanston, South Africa, were analyzed to verify the accuracy of the calibration before sample analysis, as well as throughout the analysis to monitor drift.

### 2.8. Trace Metals Analysis

Trace elements were analyzed in source water samples using an Agilent 7900 Quadrupole inductively coupled plasma mass spectrometer (ICP-MS) (Chemetix Pty Ltd., Johannesburg, South Africa). Samples were introduced via a 0.4 mL/min (7 × 10^−9^ m^3^ s^−1^) micro-mist nebulizer into a Peltier-cooled spray chamber at a temperature of 2 °C (275.15 K), with a carrier gas flow of 1.05 L/min (1.75 × 10^−5^ m^3^ s^−1^). The elements V, Cr, Mn, Fe, Co, Ni, Cu, Zn, As, Se were analyzed under He-collision mode to remove polyatomic interferences. NIST-traceable standards was used to calibrate the instrument. A NIST-traceable quality control standard of a separate supplier to the main calibration standards was analyzed to verify the accuracy of the calibration before sample analysis.

### 2.9. Anion Analysis

The anions were analyzed in source-water samples as stated in Edokpayi et al. [41]. Briefly, an Ion Chromatograph (Metrohm, Johannesburg, South Africa) was used to analyze the concentrations of fluoride, bromide, nitrates, chloride and sulfate. Calibration standards in the range of 1–20 mg/L were prepared from 100 mg/L stock solution containing all the test elements. Prior to analysis, the samples were filtered with a 0.45 μm (4.5 × 10^−7^ m) syringe filter. Eluent for the sample run was prepared from sodium bicarbonate and sodium carbonate. A 50 mmol/L sulphuric acid with a flow rate of 0.5 mL/min (8 × 10^−9^ m^3^ s^−1^) was used as suppressant.

## 3. Results

### 3.1. Socio-Demographic Characteristics of Enrolled Households

We included 405 enrolled households who completed the baseline questionnaire. The majority of caregivers were the mothers (n = 342, 84.4%, median age = 27 years) or grandmothers (n = 51, 12.6%, median age = 50 years) of a young child in the household. Almost all the caregivers had completed at least secondary school education (n = 371, 91.6%). Median monthly income for the entire household was USD$106 (interquartile range (IQR): 71–156). Access to improved sanitation was high. 373 (n = 92.1%) households used an improved pit latrine, and only 19 (n = 4.7%) reported open defecation. However, few households (n = 35,8.6%) reported having a designated place to wash hands near their toilet, and only 29% (n = 119) reported always using soap when washing hands.

Most households had their primary water source (Table 2) piped into their or their neighbor’s yard (dry: n = 226, 62.3%; wet: n = 241, 67.5%) or used a public tap (dry: n = 69, 19.0%; wet: n = 74, 20.7%). A minority (dry: n = 40, 11.0%; wet: n = 19, 5.3%) collected their water directly from rivers, lined canals, or springs. Water was collected by adult women in most households, and it was reported to take a median of 10 min (IQR, both seasons: 5–30) to go to their water source, collect water, and come back in one trip. Three quarters (n = 270, 74.4%) reported that their water source was not continually available in the dry season and two-thirds (n = 234, 65.5%) in the wet season. Almost half (48.9%) reported interruptions in availability that lasted at least 7 days in the dry season and 32.8% in the wet season. Households stored water during interruptions and/or collected water from alternative sources (dry: n = 133, 36.6%; wet: n = 115, 32.2%), which were surface water or shallow groundwater sources (e.g., rivers, lined canals, or springs).

Household water was most frequently stored in jerry cans or plastic buckets (n = 363, 89.7%), while 25 households stored water in large drums or plastic tanks (6.2%). Most households reported that their drinking water containers were covered (n = 329, 81.2%), but most used a cup with a handle (n = 281, 69.4%) or their hands (n = 93, 23.0%) for water collection (Table 3). Only 13.3% (n = 54) households reported treating their water, mainly by boiling (n = 22), chlorine (n = 15), or letting the water stand and settle (n = 11).

Approximately one-third of caregivers (n = 114, 28.2%) perceived that one can get sick from drinking water (n = 114, 28.2%), and cited diarrhea, schistosomiasis, cholera, fever, vomiting, ear infections, malnutrition, rash, flu and malaria as specific illnesses associated with water. Despite these perceptions, the majority were satisfied with their current water source (n = 297, 73.3%). Those who were unsatisfied cited reasons of insufficient quantity (n = 75), shared water supply (n = 65), uncleanliness (n = 73), cloudiness (n = 47), and bad odor or taste (n = 38).

### 3.2. Physicochemical and Microbiological Characteristics of the Water Sources

**Figure 4.**
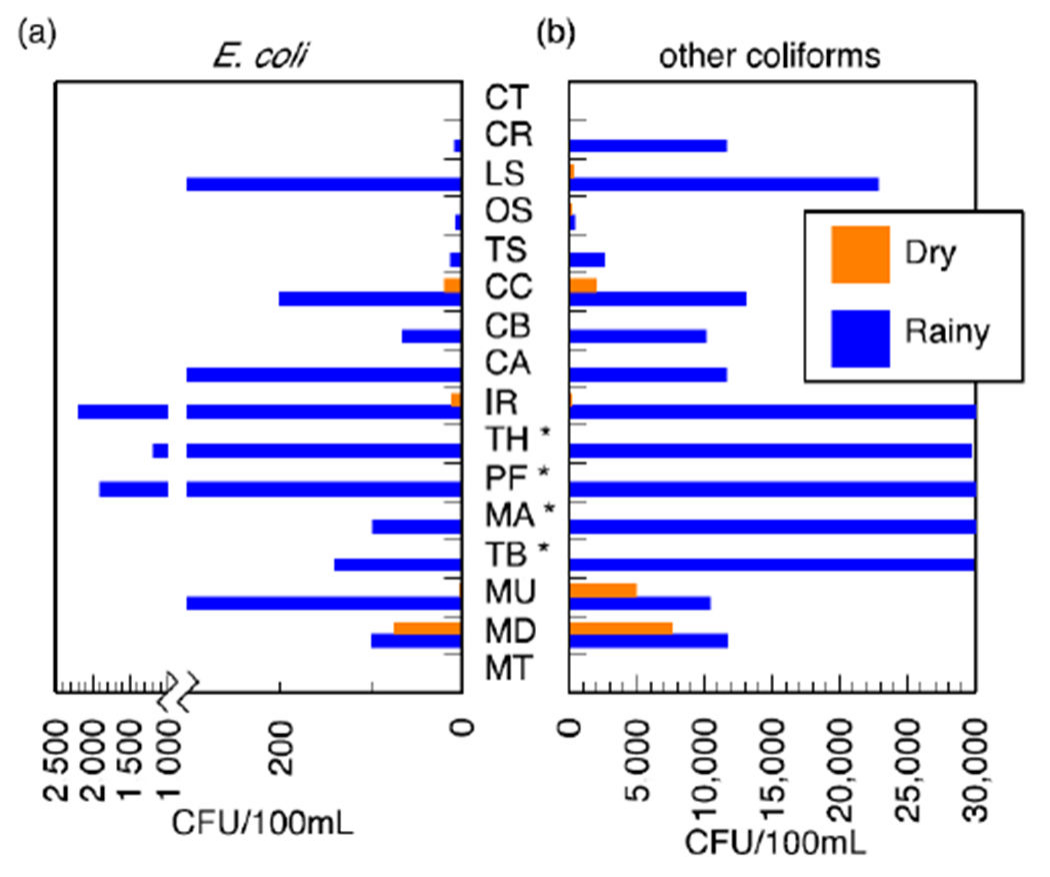
Membrane filtration results for (**a**) *E. coli* and (**b**) other coliforms. Data are presented for wet and dry seasons. The four ephemeral rivers (*) have no dry season data because they had no flow; all other sources have the results reported, some of which are zero or near-zero. South African National Standard (SANS 241:1-2015) set the limit of 0 CFU/100 mL for *E. coli* and 10 CFU/100 mL for total coliforms (CFU/10^−4^ m^3^). Ephemeral rivers that do not flow all the way into the valley are indicated (*) in the dry season.

**Figure 5.**
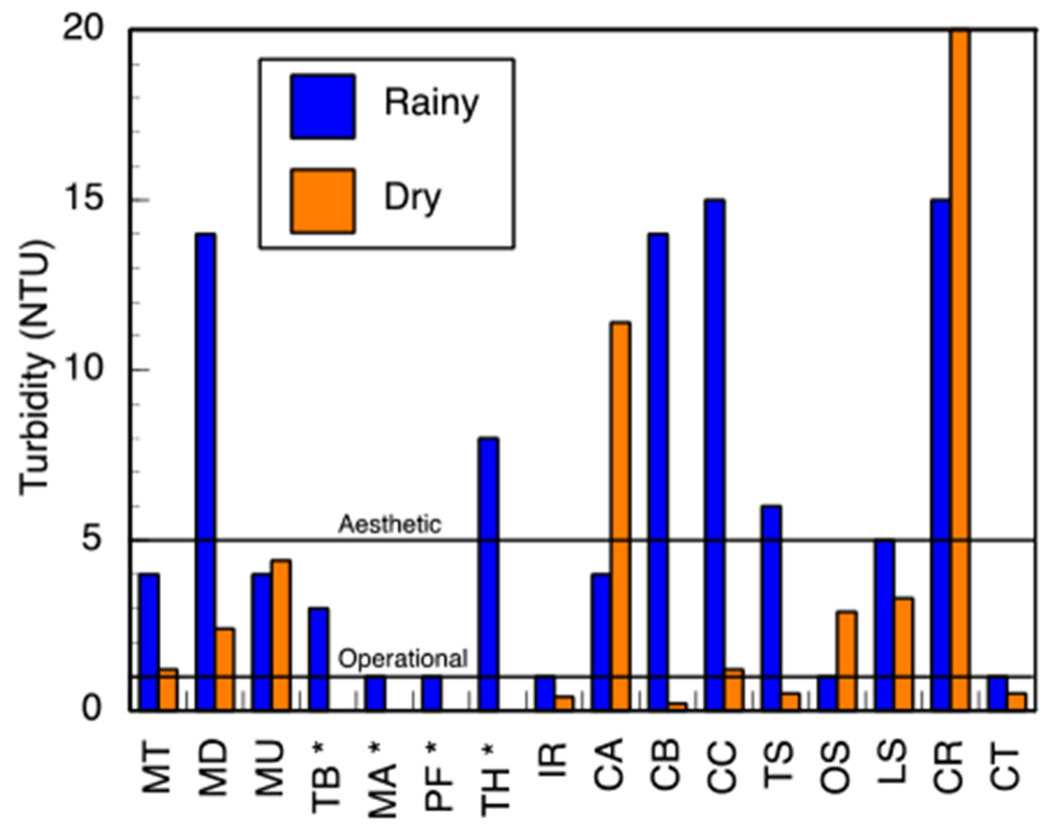
Turbidity of the water sources in the study area. Two to three measurements were taken during an intensive study period from 13 January 2017 to 4 February 2017 in the wet season and three to four measurements from 5 June 2016 to 15 July 2016 in the dry season. The median measurement of the values is reported here. Ephemeral rivers that do not flow all the way into the valley are indicated (*) in the dry season.

pH and conductivity values ranged between 5.5–7.3 and 24–405 μS/cm in the wet season and 5.8–8.7 and 8–402 μS/cm in the dry season (Table S1). Both pH and conductivity levels were within the recommended limits of the World Health Organization (WHO) for drinking water. The microbiological results and turbidity of the sources tested are presented in Figures 4 and 5, and Table S2, respectively. Microbiological data show contamination with *E. coli*, a fecal coliform that is potentially pathogenic, and other coliform bacteria.

Municipal treated water never showed any detectable colony-forming units (CFU) in a 100 mL sample for *E. coli*, which is within the Soufh African regulation [39]. In the wet season, other coliform bacteriaweae detected in the treated wtter (a median valueof 10 CFU/100 mL wac recorded).

**Figure 6.**
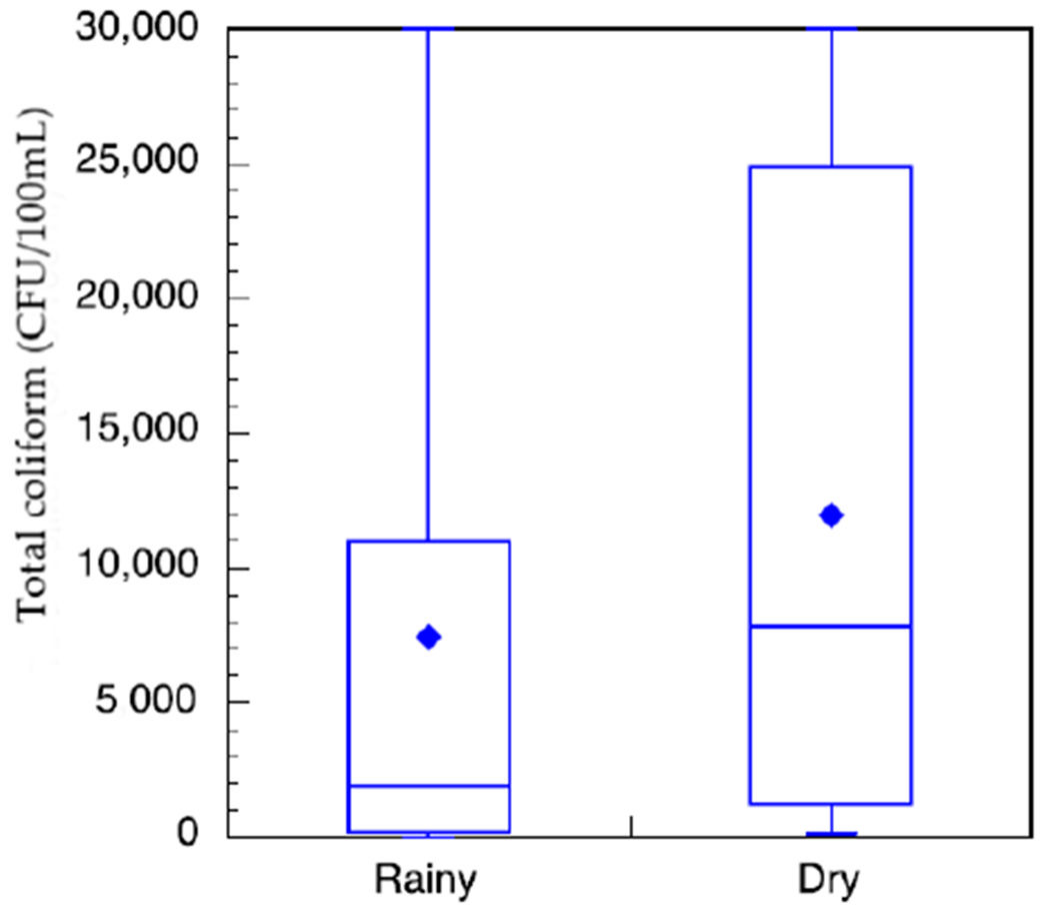
Box-and-whisker plot of total coliform measurements of stored, untreated water in study households in the wet (n = 95) and dry (n = 103) seasons. The box-and-whisker plot indicates the mean (diamond), first, second, and third quartiles (box), and minimum and maximum (whiskers).

**Figure 7.**
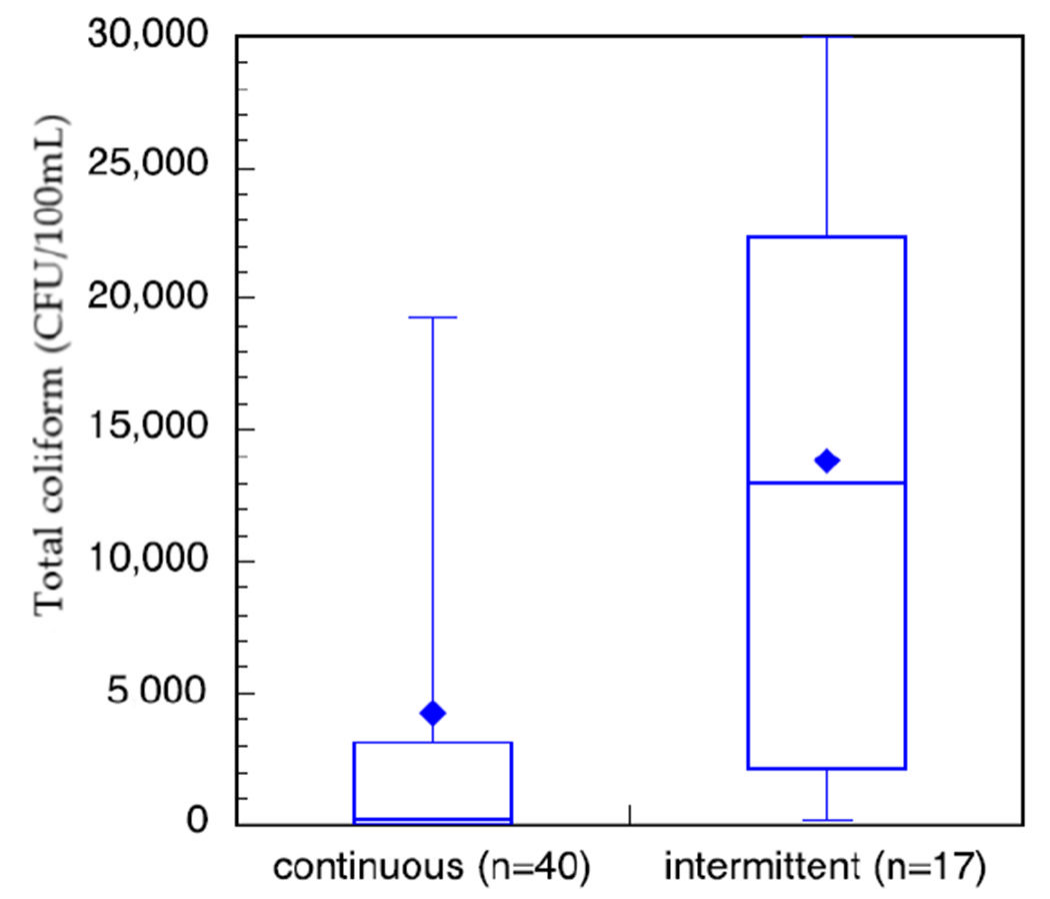
Box-and-whisker plot of total coliform measurements of stored water in the wet season in study households in communities that had verified continuous access to municipal treated water versus verified intermittent access.

Household sample of stored water (Figure 6) show that bacterial contamination levels ranged from no detectable colonies lo the maximum detection level of our protocol of 30,000 CFU/100 mL. There is a trend that total colitorm levels ere lower (during the wet season than the dry season. In the wet season, some communities within the sturdy area had access to constant municipal treated water as monitored by researcher verification of public tap-watcr availebJlity. Othet communities had intermittent access to municipal treated water. Of these honseholds, those that had constant access to treated water at or near their household did have less total coliform in their stored water than those with intermittent services (Figure 7). This neglects the communities that are outside of the municipal treated-water servic e area.

The total coliform from households in communities with verified continuous treated water had a log-normal distribution (verified by 99%, α = 1 significance level, χ^2^ goodness-of-fit test) and were statistically significantly lower (α =1 significance level) than those from households in communities with verified intermittent treated water. Unfortunately, due to the low number of samples from intermittent households, a χ^2^ goodness-of-fit test was not meaningful.

### 3.3. Anion Concentrations

Major anions investigated in the various water sources fell within the recommended guideline values from the WHO [42]. Fluoride concentrations ranged from below the detection limit (bdl) to 0.82 mg/L in the dry season and to 1.48 mg/L (Table S3) in the wet season. Fluoride levels fell below the threshold limit for fluoride in drinking water from the WHO (1.5 mg/L). Nitrates were also observed within the limit of drinking water, between bdl–17.48 mg/L and bdl–9.72 mg/L in the dry and wet seasons, respectively. Chloride, sulfate and phosphate levels were also present in moderate levels in the various water sources; however, a relatively high concentration of chloride of 462.9 mg/L was determined in the Mutale River in the wet season.

### 3.4. Trace and Major Elements Composition

**Figure 8.**
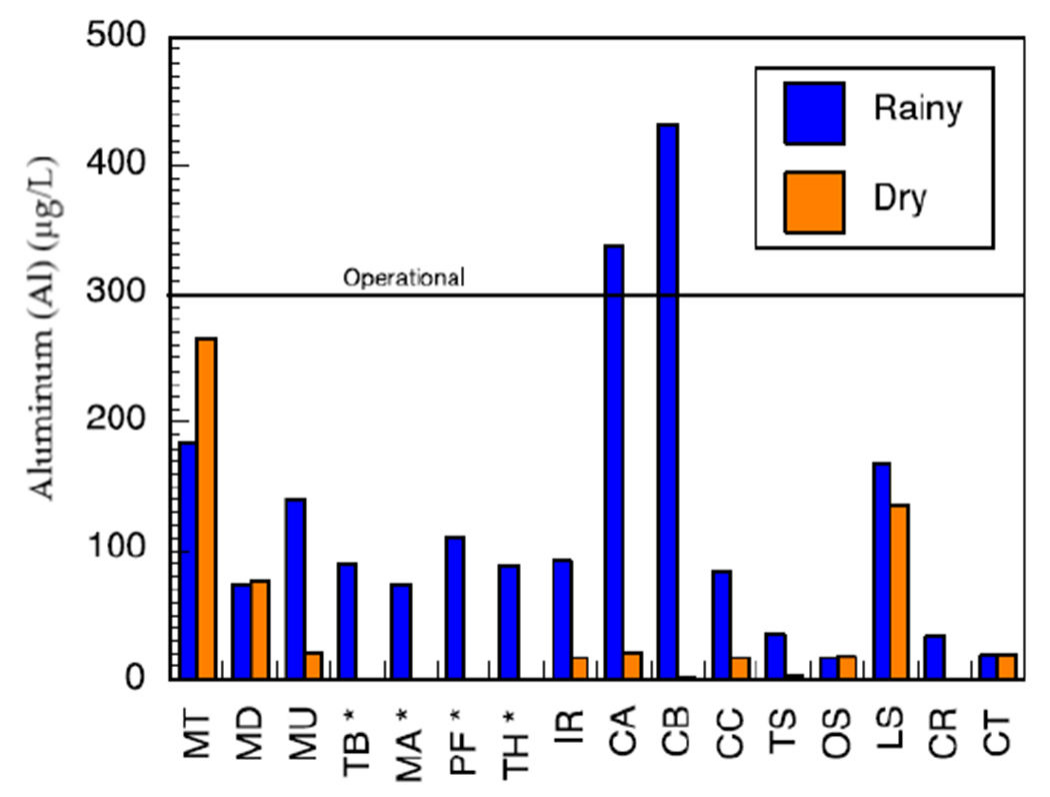
Aluminum, measured by an inductively coupled plasma mass spectrometer (ICP-MS), concentration for natural sources in the study area in the wet and dry seasons. The SANS 241 standard is shown (an operational standard is intended for treated water). Sources marked with * are intermittent sources and had no dry-season sample. Other sources have measured concentrations; although they may be too low to plot.

**Figure 9.**
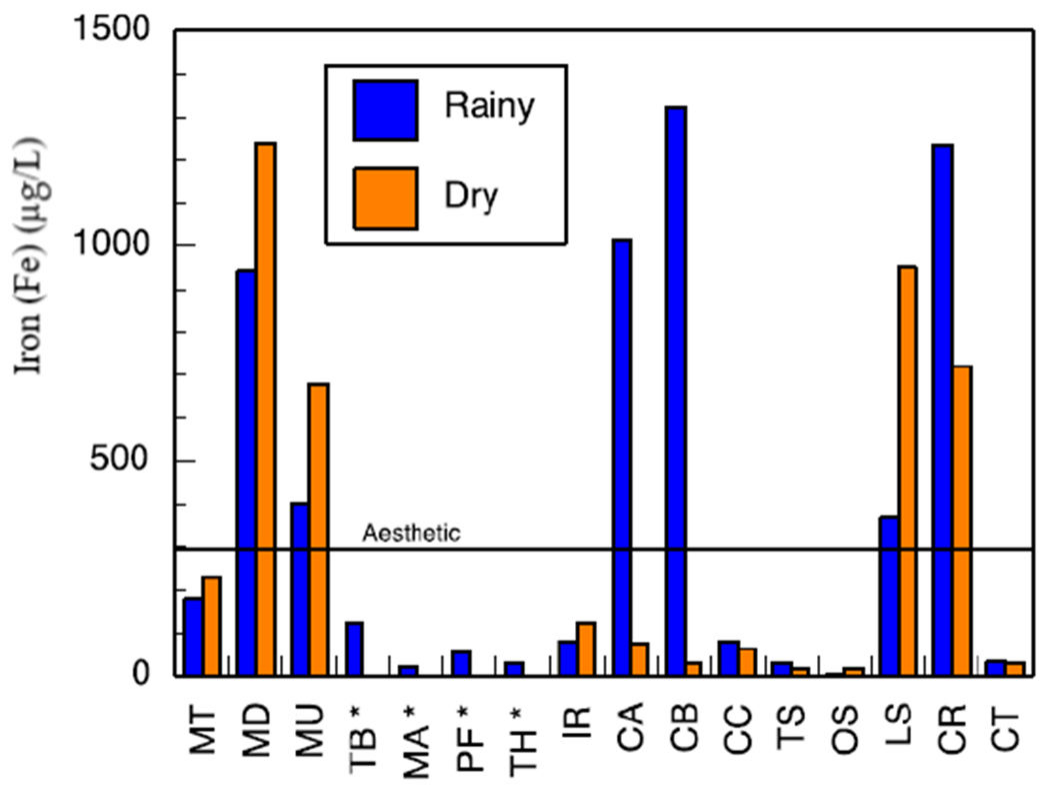
Iron, measured by an ICP-MS, concentration for natural sources in the study area in the wet and dry seasons. The SANS 241 standard is shown. Sources marked with * are intermittent sources and had no dry-season sample. Other sources have measured concentrations; although they may be too low to plot.

Major metals in the various water sources in both seasons complied with the recommended limits of SANS and WHO in drinking water [39,42]. Sodium concentrations in the range of 3.14–41.03 mg/L and 3.02–15.34 mg/L were measured in the wet and the dry seasons, respectively (Table S4). Low values of potassium were measured. Calcium levels ranged between 0.66–33.91 mg/L and 0.53–27.39 mg/L, in the wet and dry seasons, respectively. Low levels of magnesium were also found. Most of the water sources can be classified as soft water owing to the low levels of calcium and magnesium. Aluminium (Al) concentration ranged between 39.18–438 μg/L (Figure 8). Two of the water sources which are community-based water supply systems recorded high levels of Al which exceeded the aesthetic permissible levels of drinking water; others fell within this limit. Similarly, the levels of iron (Fe) varied between 37.30–1354 mg/L and 35.21–1262 mg/L in the wet and the dry seasons, respectively (Figure 9). Some of the sources showed high Fe concentration which exceeded the aesthetic permissible limit of WHO in drinking water [42]. Two community-based water systems had higher levels of Fe in the wet season as well as the major river in the region (Mutale River) for which high Fe levels were observed in both seasons. One of the clinic boreholes also recorded high levels of Fe above the permissible aesthetic value of (300 mg/L) in both seasons. Temporary seasonal variation was significant only in the levels of Fe and Al. In the wet season, their levels were generally higher than in the dry season. Some other trace metals of concern like Pb, Hg, As, Cd, Cr, Ni, Cu, Mn, Sr were all present at low levels that were below their recommended limits in drinking water for both seasons (Table S5).

## 4. Discussion

This study provides a comprehensive description of water quality and drinking-water use across seasons in a low-resource community in rural South Africa, including a variety of water sources, ranging from the municipal tap to natural sources and a combination of both when the municipal tap was intermittently available.

Water sources in the study area, aside from the municipal tap, were highly contaminated with *E. coli* in both the wet and dry seasons; that is, *E. coli* was above the South African standard (acute health) of 0 CFU/100 mL. It is particularly important to note that *E. coli* was detected in the boreholes used for water at the local clinics, implying inadequate access to potable water for potentially immunocompromised patients. While the municipal treated water met the *E. coli* detection limit, the municipal tap did not always fall within the standards of turbidity (≤1 NTU operational and ≤5 NTU aesthetic) and total coliform (≤10 CFU/100 mL) [39]. These are not direct health risks; however, both measurements can be used to judge the efficacy of the treatment process and suggest that treatment may not have removed other pathogens that were not directly tested, such as protozoan parasites.

While the microbiological contamination of the drinking-water sources was not acceptable, the chemical constituents fell within the South African guidelines [39]. Calcium, sodium, magnesium and potassium were present in low levels and their concentrations complied with regulatory standards of SANS [39] and WHO [42]. Some metals (cadmium, mercury, arsenic and lead) known to be carcinogenic, mutagenic and teratogenic, causing various acute and chronic diseases to humans even at trace levels in drinking water, were investigated and found to be present in very low concentrations that could be of no health risk to the consumers of the various water resources in the region. However, some other metals, such as Al and Fe, were higher in some of the water sources; yet these were still well below the health guidelines for the respective constituent (recommended health levels from SANS and WHO are given as Al < 0.9 mg/L, Fe < 2 mg/L). At these levels, they do not present a health risk but could impart color and significant taste to the water thereby affecting its aesthetic value. Water sources from the community water-supply systems and one of the clinic boreholes recorded higher levels of Al and Fe. The other metals evaluated (copper, zinc, nickel, chromium, Se and Mn) were present in low levels that complied with their recommended limits in drinking water [39,42].

Fluoridation of drinking water is a common practice for oral health in many countries [43]. The required level of fluoride to reduce incidences of dental caries is in the range of 0.6–0.8 mg/L; however, levels above 1.5 mg/L are associated with dental and skeletal fluorosis [43–45]. The likelihood of fluorosis as a result of high concentration of fluoride is low in these communities, but there could be a high incidence of dental caries since fluoride levels below 0.6 mg/L were measured and some of the water sources did not have fluoride concentrations detectable by the instrument. The National Children’s Health Survey conducted in South Africa showed that 60.3% of children in the age group of 6 years have dental caries. Approximately a third (31.3%) of children aged 4–5 years in Limpopo province have reported cases of dental caries [44,45].

Chloride levels in the water sources do not cause any significant risk to the users except imparting taste to the water for some of the sources that recorded chloride levels above 300 mg/L. Although the study area is characterized by farming activities, the nitrate concentrations measured do not present any health risks. Therefore, the occurrence of methemoglobinemia or blue-baby syndrome as a result of high nitrate levels is unlikely. Other anions were present in moderate levels that would also not constitute any health risks. The levels of all the anions determined in the various sources were lower than the recommended guidelines of WHO [42].

The microbiological analysis of environmental water sources revealed several trends. Without exception in these samples, bacterial levels in the wet season were higher than in the dry season. This may be caused by greater runoff or infiltration, which carries bacteria from contaminated sources to these water bodies. The upward trend in bacteria in the municipal treated water is not explained by an increase in runoff, but may be due to higher turbidity of the intake for the municipal treated water in the wet season. The treatment facility workers reported to the researchers that they were unable to monitor the quality of the treated water due to instrument failure during the wet season surveillance period.

Water stored in the household showed that the mean total coliform in the wet season was lower than that in the dry season. This trend is opposite to what was observed in the source, or environmental samples. This difference may be explained by the greater availability of treated water in the wet season versus the dry season for approximately 40% of the sampled households (Figure 7). In addition, it is possible that families try to save water during the dry season and do not reject residual water, while the rainy season allows easier washing of the container and for it to be filled with fresh water more regularly.

In the wet season, two communities had consistently treated water available from household connections (usually a tap somewhere in a fence-in yard) or public taps. While the municipal treated water was of lower quality in the wet season than the dry season, the quality was significantly better than most environmental sources.

Another potential explanation is that residents stored their water within their households for a shorter time, which is supported by the use data that showed interruptions in supply were more common and for longer duration in the dry season. The quality of the water stored in households with continuous supply versus intermittent supply also suggests that water availability may play a role in household water quality. This is consistent with research that demonstrates that intermittent water supply introduces contamination into the distribution system in comparison with continuous supply [46]. Intermittent supply of water may also result in greater quantity and duration of storage at household level, which could increase the likelihood of contamination.

While it has been shown that the quality of water used for drinking in these villages does not meet South African standards, this problem is confounded by evidence from surveys indicating that residents believe they have high-quality water and, therefore, do not use any form of treatment. In the rare case that they do, it is by letting the water stand and settle or by boiling. In addition, even if treated water is collected, there is a risk of recontamination during storage and again when using a cup held by a hand to retrieve water from storage devices, which was common in surveyed homes. In addition, there was little to no detectable residual chlorine in the municipal tap water to prevent recontamination. A previous study performed in an adjacent community showed higher household treatment levels; however, this may have been due to intervention studies in that community (the community in question was excluded from this study because of previous interventions) [47]. The study also concurred that boiling was the most common method employed.

Given that most of the water from the various sources in this community is contaminated and not treated, there is a high risk of enteric disease in the community. Lack of access to adequate water and sanitation cause exposure to pathogens through water, excreta, toxins, and water-collection and storage pathways, resulting in immense health impacts on communities [48]. A large burden of death and disability due to lack of access to clean water and sanitation is specifically associated with diarrheal diseases, intestinal helminths, schistosomiasis and trachoma [49]. While it was found in this study that the study area has a high prevalence of improved sanitation, the likelihood of poor water quality due to intermittent supply and lack of treatment poses a risk of the adverse health effects described. In a previous longitudinal cohort study of children in these villages, most children were exclusively breastfed for only a month or less, and 50% of children had at least one enteropathogen detected in a non-diarrheal stool by three months of age [50]. Furthermore, the burden of diarrhea was 0.66 episodes per child-year in the first 2 years of life, and stunting prevalence (length-for-age z-score less than −2) in the cohort increased from 12.4% at birth to 35.7% at 24 months [50]. It is likely that contaminated water contributed to the observed pathogen burden and stunting prevalence in these communities. In summary, microbiological contamination of the drinking water is high in the study area, and risk from other chemical constituents is low. Therefore, engineered solutions should focus more on improving the microbiological quality of the drinking water.

The intermittent supply in municipal tap water, inadequate water quality from alternative sources, and the risk of recontamination during storage suggest a need for a low-cost, point-of-use water-treatment solution to be used at the household level in these communities. Access to clean drinking water will contribute to improving the health of young children who are at highest risk of the morbidity and mortality associated with waterborne diseases. Such an intervention may go beyond the prevention of diarrhea by impacting long-term outcomes such as environmental enteropathy, poor growth and cognitive impairment, which have been associated with long-term exposure to enteropathogens [51]. This is supported by a recent finding that access to improved water and sanitation was associated with improvements on a receptive vocabulary test at 1, 5 and 8 years of age among Peruvian, Ethiopian, Vietnamese and Indian children [52]. The implementation of point-of-use water treatment devices would ensure that water is safe to drink before consumption in the homes of these villages, improving child health and development.

## 5. Conclusions

This study was comprehensive in the assessment of all aspects of water quality and corresponding water-use practices in rural areas of Limpopo Province. The results obtained indicate that microbiological water quality is more likely to have adverse effect on the consumers of natural water without adequate treatment, as *E. coli* was determined in all the natural water sources. Local needs assessments are critical to understanding local variability in water quality and developing appropriate interventions. Interventions to ensure clean and safe drinking water in rural areas of Limpopo province should, first and foremost, consider microbiological contamination as a priority. Risk-assessment studies of the impact of water quality on human health is, therefore, recommended.

## Supplementary Material

Tables S1 through S5 Table S1: Physical characteristics of water sources. Two to three measurements were taken during an intensive study period from 13 January 2017 to 4 February 2017 in the wet season, and three to four measurements from 5 June 2016 to 15 July 2016 in the dry season. The median measurement of the values is reported here. Sites with missing samples, such as ephemeral rivers that do not flow all the way into the valley in the dry season, are indicated (*). Sites with missing data due to instrument failure are indicated (#). Values that were below the detection limit are indicated (bdl). South African regulation (SANS 241:1-2015) and the World Health Organization Recommended Guidelines for Drinking Water Quality (Fourth Edition) are listed; parameters not listed are indicated (nl),
Table S2: Membrane-filtration results for E. Coli and total coliforms of water sources,
Table S3: Anion concentrations (mg/L) of water sources,
Table S4: Major metal concentrations (mg/L) of water sources,
Table S5: Trace metal concentrations μg/L) of water sources.

## Figures and Tables

**Table 1.** Abbreviations, water sources and type.

Source Name	Abbreviation	Type
Municipal Tap (Branch 2)	MT	treated
Mutale River-downstream	MD	surface
Mutale River-upstream	MU	surface
Tshiombedi River	TB	surface
Madade River	MA	surface
Pfaleni River	PF	surface
Tshala River	TH	surface
Irrigation Canal (Tshala River diversion)	IR	diversion (from surface)
Community A System	CA	diversion (from surface)
Community B System	CB	diversion (from surface)
Community C System	CC	diversion (from surface)
Tshibvumo/Mbahela Spring	TS	shallow groundwater
Mbulugeni’s Orchard Spring	OS	shallow groundwater
Lutsingeni Spring	LS	shallow groundwater
Clinic A Borehole	CR	groundwater
Clinic B Borehole	CT	groundwater
Madadani Borehole	MB	groundwater
Pile Spring	PS	shallow groundwater

**Table 2.** Primary drinking-water sources reported among 363 and 357 households in the study area in the dry and wet seasons, respectively.

Main Drinking-Water Source	Number (%)	
	Dry Season	Wet Season

	n = 363	n = 357
Piped into house	13 (3.6)	15 (4.2)
Piped into yard	189 (52.1)	218 (61.1)
Neighbor’s pipe	37 (10.2)	23 (6.4)
Public tap	69 (19.0)	74 (20.7)
Natural source	40 (11.0)	19 (5.3)
Tanker truck	0 (0.0)	0 (0.0)
Other	15 (4.1)	8 (2.2)

**Table 3.** Mode of water collection from storage containers.

Mode of Water Collection	n (%)
Pour directly	21 (5.2)
Use cup with handle	281 (69.4)
Use cup with hands	93 (23.0)
Spigot	4 (1.0)
Other	6 (1.5)
