# Supplementary material for: Challenges to Sustainable Safe Drinking Water: A Case Study of Water Quality and Use across Seasons in Rural Communities in Limpopo Province, South Africa
Source: Water (Basel). Author manuscript; Available in PMC 2018 Dec 28. (PMC6310213; doi:10.3390/w10020159)
Supplement: Tables S1 through S5 — Table S1: Physical characteristics of water sources. Two to three measurements were taken during an intensive study period from 13 January 2017 to 4 February 2017 in the wet season, and three to four measurements from 5 June 2016 to 15 July 2016 in the dry season. The median measurement of the values is reported here. Sites with missing samples, such as ephemeral rivers that do not flow all the way into the valley in the dry season, are indicated (*). Sites with missing data due to instrument failure are indicated (#). Values that were below the detection limit are indicated (bdl). South African regulation (SANS 241:1-2015) and the World Health Organization Recommended Guidelines for Drinking Water Quality (Fourth Edition) are listed; parameters not listed are indicated (nl), Table S2: Membrane-filtration results for E. Coli and total coliforms of water sources, Table S3: Anion concentrations (mg/L) of water sources, Table S4: Major metal concentrations (mg/L) of water sources, Table S5: Trace metal concentrations μg/L) of water sources. [file NIHMS989490-supplement-Tables_S1_through_S5.pdf]

S1 Table: Physical characteristics of a range of water sources in the study area. Two to three measurements were taken during an intensive operation period from 13 January 2017 to 04 February 2017 in the wet season and three to four measurements from 05 June 2016 to 15 July 2016 in the dry season. The median measurement of the values is reported here. Ephemeral rivers that do not flow all the way into the valley are indicated (*) in the dry season.

Sources	Wet pH	Dry pH	Wet Turbidity (NTU)	Dry Turbidity (NTU)	Wet EC (µs/cm)	Dry EC (µs/cm)
MT	7.3	8.7	4	1	47	70
MD	6.9	8.6	14	2	34	62
MU	6.9	5.9	4	4	23	55
TB	5.6	*	3	*	26	*
MA	5.5	*	1	*	27	*
PF	6.0	*	1	*	24	*
TH	6.4	*	8	*	30	*
IR	7.2	6.7	1	0	29	40
CA	6.4	5.8	4	11	24	8
CB	6.4	7.2	14	0	24	68
CC	6.8	6.4	15	1	74	77
TS	6.1	6.5	6	1	104	120
OS	5.6	6	1	3	69	73
LS	6.3	7.1	5	3	82	103
CR	6.7	6.9	15	21	204	285
CT	7.1	7.3	1	1	405	402
MB	7.5	*	111	*	163	*
PS	*	5.4	*	1	*	54
WHO	nl	nl	nl	nl	nl	nl
SANS	5 to 9.7 (operational)	5 to 9.7 (operational)	1 (operational) 5 (aesthetic)	1(operational) 5 (aesthetic)	17000 (aesthetic)	17000 (aesthetic)

S2 Table: Membrane filtration results for *E. coli* and total coliforms. South African regulation (SANS 241:1-2015) set the limit of 0 CFU/100 mL for *E. coli* and 10 CFU/100 mL for total coliforms. Ephemeral rivers that do not flow all the way into the valley are indicated (*) in the dry season.

Sources	Wet<br>E. coli<br>(cfu/100 mL)	Dry<br>E. coli<br>(cfu/100 mL)	Wet<br>Total Coliform<br>(cfu/100 mL)	Dry<br>Total coliform<br>(cfu/100 mL)
MT	0	0	10	0
MD	100	74	11800	7600
MU	300	3	10700	5000
TB	140	*	30000	*
MA	98	*	30000	*
PF	1900	*	31900	*
TH	1200	*	31000	*
IR	2200	11	32200	178
CA	300	1	11900	89
CB	66	0	10100	45
CC	200	18	13200	2000
TS	12	0	2600	49
OS	6	0	400	130
LS	800	0	23600	271
CR	7	0	11600	2
CT	0	0	152	52
MB	200	*	30000	*
PS	*	0	*	42
WHO	0	0	indicator only	indicator only
SANS	0<br>(acute health)	0<br>(acute health)	10<br>(operational)	10 (operational)

S3 Table: Anions concentration (mg/L) in various water sources within the study area, bdl and NA, NQ represent below detection limit, no available data and not quantified. Ephemeral rivers that do not flow all the way into the valley are indicated (*) in the dry season. Sources: WHO and SANS represent, World Health Organization and South African National Standards.

Sources	Anions (mg/L)										
	F^- wet	F^- dry	Br^- wet	Br^- dry	SO_4^{2-} wet	SO_4^{2-} dry	NO_3^- wet	NO_3^- dry	PO_4^{3-} wet	PO_4^{3-} dry	Cl^- wet	Cl^- dry
MT	1.33	bdl	5.32	11.09	2.8	bdl	bdl	bdl	bdl	3.95	21.3	58.57
MD	1.29	bdl	bdl	5.41	3.35	bdl	bdl	bdl	bdl	bdl	10.93	36.99
MU	#	bdl	#	4.84	#	bdl	#	bdl	#	bdl	#	31.54
TB	1.33	*	#	*	2.95	*	bdl	*	3.92	*	bdl	*
MA	1.31	*	#	*	3.63	*	bdl	*	bdl	*	19.24	*
PF	1.29	*	3.23	*	3.79	*	bdl	*	bdl	*	23.04	*
TH	1.3	bdl	4.46	4.86	3.65	0.63	bdl	bdl	bdl	bdl	20.65	33.67
IR	1.29	bdl	3.85	6.54	3.86	0.82	bdl	bdl	bdl	0.56	19.95	34.43
CA	1.29	bdl	2.09	11.83	3.05	bdl	bdl	bdl	bdl	1.78	10.09	54.99
CB	#	bdl	#	5.11	#	bdl	#	bdl	#	bdl	#	53.72
CC	1.29	bdl	1.99	7.61	3.33	bdl	bdl	bdl	bdl	bdl	9.99	40.93
TS	1.3	#	4.08	#	2.51	#	9.72	#	bdl	#	28.25	#
OS	1.29	0.47	2.25	10.06	3.3	1.3	5.56	bdl	bdl	3.25	12.78	94.35
LS	1.29	0.82	3.33	24.19	3.88	1.65	5.21	bdl	bdl	7.86	11.27	85.15
CR	#	0.33	#	4.96	#	0.9	#	2.47	#	0	#	52.56
CT	1.48	*	1.35	*	2.69	*	5.35	*	3.99	*	10	*
MB	#	*	#	*	#	*	#	*	#	*	#	*
PS	*	bdl	*	1.93	*	bdl	*	0.64	*	bdl	*	44.63
WHO	1.5	1.5	nl	nl	nl	nl	50	50	nl	nl	nl	nl
SANS	1.5	bdl	nl	11.09	250	250	50	50	nl	nl	300	300

S4 Table: Major metal concentrations (mg/L) in various water sources around the study area, where bdl represent below detection limit. WHO and SANS represent, World Health Organization and South African National Standards. NA represent no available date.

Sources	Metals (mg/L)													
	Na wet	Na dry	Ca wet	Ca dry	K wet	K dry	Mg wet	Mg dry	S^{2-} wet	S^{2-} dry	Si wet	Si dry	P wet	P dry
MT	4.56	5.66	5.16	5.16	0.32	1.07	1.27	2.92	0.37	0.34	2.92	4.33	bdl	0.04
MD	4.46	5.78	2.15	3.16	0.35	0.27	1.67	2.8	0.3	0.27	3.61	4.39	bdl	0.01
MU	3.08	4.54	1.44	3.34	0.6	0.08	1	2.97	0.34	0.25	2.16	3.58	0.02	0
TB	3.79	*	0.72	*	0.52	*	0.56	*	0.28	*	1.88	*	bdl	*
MA	4.15	*	0.6	*	0.46	*	0.63	*	0.24	*	1.87	*	bdl	*
PF	2.84	*	0.66	*	0.59	*	0.51	*	0.29	*	1.29	*	bdl	*
TH	4.55	*	1.04	*	0.81	*	1.02	*	0.34	*	2.15	*	bdl	*
IR	4.46	3.47	1.07	0.65	0.98	0.09	0.94	1.35	0.33	0.18	2.07	3.25	bdl	0
CA	4.09	2.96	1.35	0.49	0.61	0.03	0.86	0.69	0.26	0.12	2.42	3.07	0.02	0
CB	6.07	4.6	1.83	3.03	0.17	0.18	4.21	5.36	0.2	0.08	6.08	5.18	0.01	0.01
CC	3.27	4.92	0.59	4.81	0.16	0.04	0.64	2.31	0.15	0.4	2.08	5.14	bdl	0.01
TS	6.78	6.24	7.05	6.51	0.13	bdl	4.49	4.86	0.1	0.08	12.13	12.14	0.01	0.01
OS	5.53	5.67	2.9	2.76	0.03	bdl	3.2	3.22	0.04	0.04	4.12	4.13	bdl	0
LS	7.76	7.08	5.13	3.53	0.4	0.02	3.91	3.04	0.12	0.15	10.65	9.83	bdl	0.01
CR	13.9	12.7	18.93	18.23	0.11	0.06	14.59	15.5	0.78	0.41	18.12	17.06	bdl	0.01
CT	41.0	36.2	33.83	33.43	1.22	1.04	6.8	7.45	0.52	0.55	15.82	16.77	0.12	0.13
MB	16.2	*	19.91	*	0.4	*	13.71	*	0.23	*	22.5	*	0.01	*
PS	*	7.13	*	1.09	*	0.18	*	1.07	*	0.15	*	5.46	*	0.01
WHO	nl	nl	nl	nl	nl	nl	nl	nl	nl	nl	nl	nl	nl	nl
SANS	200	200	nl	nl	nl	nl	nl	nl	nl	nl	nl	nl	nl	nl

S5 Table: Trace metal concentrations (μg/L) in various water sources around the area of study, where bdl and NA represent below detection limit and No available guideline value. WHO and SANS represent, World Health Organization and South African National Standards.

Metals (μg/L)	CR dry	CR wet	CT dry	CT wet	MU dry	MU wet	MD dry	MD wet	IR dry	IR wet	PS dry	PS wet	WHO [21]	SANS [18]
Li	0.73	1.53	2.70	1.21	0.19	bdl	bdl	0.14	0.20	bdl	bdl	bdl	NA	NA
Be	0.01	bdl	bdl	0.05	bdl	bdl	0.02	bdl	bdl	0.00	0.02	0.02	NA	NA
B	19.14	13.48	13.79	14.44	8.05	6.58	8.18	8.56	5.71	9.16	5.68	9.38	NA	2400
Al	5.12	39.18	5.56	25.10	27.93	145.67	80.42	83.62	23.51	97.83	34.84	80.02	NA	300
V	4.78	6.72	8.87	0.81	0.71	0.78	0.98	0.76	0.33	0.47	0.37	0.30	NA	100
Cr	3.63	3.91	3.84	3.63	3.93	4.27	4.22	4.10	3.83	6.70	3.80	5.63	50	50
Mn	5.69	8.37	1.48	2.55	56.20	41.67	76.05	52.89	3.04	4.44	27.28	12.80	NA	100/400
Fe	741.75	1268.27	87.91	65.00	1262.11	436.00	975.25	696.56	142.50	113.94	446.84	85.71	NA	300/2000
Co	0.05	0.15	0.04	0.09	0.60	0.68	0.81	0.52	0.13	0.31	2.26	0.34	NA	NA
Ni	3.29	3.95	2.03	2.89	2.21	3.85	3.08	5.84	2.57	5.53	4.92	4.20	70	70
Cu	55.35	12.15	0.25	13.02	0.14	7.94	3.95	15.60	2.06	9.84	4.21	5.35	2000	2000
Zn	177.15	61.32	13.81	67.90	0.96	69.07	35.54	13.47	3.47	67.10	4.20	35.74	NA	5000
As	0.10	1.20	0.15	1.33	0.06	1.33	0.57	0.08	0.04	0.39	0.05	0.13	10	10
Se	0.11	0.08	0.11	0.06	0.03	0.05	0.06	0.02	0.02	0.03	0.02	0.04	40	40
Sr	30.80	35.00	57.49	93.79	12.19	6.10	10.99	15.46	5.98	6.70	12.85	7.26	NA	NA
Mo	0.14	0.14	0.16	0.34	0.12	0.15	0.14	0.14	0.12	0.15	0.13	0.11	NA	NA
Cd	0.05	0.02	0.00	0.04	bdl	0.06	0.02	0.03	0.00	0.11	0.01	0.06	3	3
Sb	0.01	0.25	0.02	0.21	0.00	1.02	0.78	0.25	0.03	0.64	0.07	0.18	20	20
Hg	0.01	0.01	0.01	0.02	0.01	0.00	0.00	0.01	0.01	0.04	0.01	0.03	6	6
Pb	1.31	1.33	0.23	1.40	0.20	1.61	0.76	0.91	0.41	1.58	0.39	0.89	10	10
Ba	bdl	8.25	bdl	47.23	bdl	7.60	9.81	0.01	bdl	7.47	0.02	9.91	700	700

S5 Table continued

Metals (µg/L)	CA dry	CA wet	CB dry	CB wet	CC dry	CC wet	TS dry	TS wet	MB wet	MT dry	MT wet	WHO [21]	SANS [18]
Li	0.24	bdl	bdl	bdl	bdl	bdl	0.79	bdl	1.48	0.20	bdl	NA	NA
Be	bdl	0.05	0.01	0.00	bdl	0.00	0.01	0.05	0.02	bdl	0.00	NA	NA
B	4.59	8.84	5.01	4.69	2.99	6.33	8.00	6.19	14.50	9.95	7.02	NA	2400
Al	27.81	344.28	8.40	437.95	23.40	90.51	9.79	40.54	113.91	271.25	0.69	NA	300
V	0.23	0.97	0.96	8.21	1.40	0.92	0.74	1.08	10.57	2.58	0.57	NA	100
Cr	3.76	4.60	3.83	5.21	3.82	4.33	3.95	3.97	3.90	4.62	4.06	50	50
Mn	2.69	29.63	3.30	50.55	2.21	3.63	1.19	2.80	33.23	17.92	45.68	NA	100/400
Fe	96.64	1046.87	48.66	1354.28	81.88	115.09	35.21	63.93	126.48	253.30	210.12	NA	300/2000
Co	0.11	0.88	0.08	1.71	0.11	0.18	0.04	0.11	0.55	1.07	0.21	NA	NA
Ni	2.85	4.16	3.09	4.76	2.96	2.66	3.03	3.33	3.45	9.83	2.78	70	70
Cu	2.01	8.47	0.12	4.82	1.81	2.27	1.55	2.93	1.99	75.31	3.70	2000	2000
Zn	3.51	112.42	2.14	44.88	3.09	34.14	2.17	63.79	1955.39	88.08	40.73	NA	5000
As	0.03	0.68	0.03	0.57	0.13	0.55	0.05	2.57	0.49	0.19	0.69	10	10
Se	0.01	0.06	0.02	0.04	0.04	0.05	0.03	0.04	0.04	0.02	0.05	40	40
Sr	3.58	5.47	15.77	12.18	24.75	4.41	23.90	23.40	51.64	19.65	18.12	NA	NA
Mo	0.12	0.14	0.14	0.13	0.20	0.13	0.15	0.13	0.17	0.16	0.15	NA	NA
Cd	0.00	0.09	0.00	0.03	0.01	0.02	0.00	0.03	0.01	0.09	0.02	3	3
Sb	0.05	0.24	0.00	0.22	0.04	0.13	0.02	0.49	0.21	0.38	6.36	20	20
Hg	0.02	0.01	0.01	0.01	0.01	0.01	0.01	bdl	bdl	0.02	bdl	6	6
Pb	0.35	1.64	0.24	0.99	0.34	0.54	0.28	1.01	0.57	3.79	0.25	10	10
Ba	bdl	7.52	0.01	13.59	bdl	5.03	0.01	8.63	10.79	0.01	0.78	700	700
